# Case report: High-frequency repetitive transcranial magnetic stimulation for treatment of hereditary spastic paraplegia type 11

**DOI:** 10.3389/fneur.2023.1162149

**Published:** 2023-05-18

**Authors:** Songmei Chen, Zhiqing Zhou, Meng Ren, Xixi Chen, Xiaolong Shi, Sicong Zhang, Shutian Xu, Xiaolin Zhang, Xingyuan Zhang, Wanlong Lin, Chunlei Shan

**Affiliations:** ^1^Department of Rehabilitation Medicine, Shanghai No. 3 Rehabilitation Hospital, Shanghai, China; ^2^School of Rehabilitation Science, Shanghai University of Traditional Chinese Medicine, Shanghai, China; ^3^Center of Rehabilitation Medicine, Yueyang Hospital of Integrated Traditional Chinese and Western Medicine, Shanghai University of Traditional Chinese Medicine, Shanghai, China; ^4^Engineering Research Center of Traditional Chinese Medicine Intelligent Rehabilitation, Ministry of Education, Shanghai, China

**Keywords:** hereditary spastic paraplegia, repetitive transcranial magnetic stimulation, diffusion tensor imaging, corticospinal tract, lower extremity spasticity, walking ability

## Abstract

Hereditary spastic paraplegia (HSP) is a heterogeneous group of inherited neurodegenerative disorders that currently have no cure. HSP type 11 (SPG11-HSP) is a complex form carrying mutations in the SPG11 gene. Neuropathological studies demonstrate that motor deficits in these patients are mainly attributed to axonal degeneration of the corticospinal tract (CST). Repetitive transcranial magnetic stimulation (rTMS) is a non-invasive technique that can induce central nervous system plasticity and promote neurological recovery by modulating the excitability of cortical neuronal cells. Although rTMS is expected to be a therapeutic tool for neurodegenerative diseases, no previous studies have applied rTMS to treat motor symptoms in SPG11-HSP. Here, we report a case of SPG11-HSP with lower extremity spasticity and gait instability, which were improved by applying high-frequency rTMS (HF-rTMS) at the primary motor cortex (M1). Clinical and physiological features were measured throughout the treatment, including the Modified Ashworth Scale (MAS), Berg Balance Scale (BBS), the timed up and go (TUG) test and the 10-meter walk test time (10 MWT). The structure and excitability of the CST were assessed by diffusion tensor imaging (DTI) and transcranial magnetic stimulation (TMS), respectively. After treatment, the patient gained 17 points of BBS, along with a gradual decrease in MAS scores of the bilateral lower extremity. In addition, the TUG test and 10 MWT improved to varying degrees. TMS assessment showed increased motor evoked potential (MEP) amplitude, decreased resting motor threshold (RMT), decreased central motor conduction time (CMCT), and decreased difference in the cortical silent period (CSP) between bilateral hemispheres. Using the DTI technique, we observed increased fractional anisotropy (FA) values and decreased mean diffusivity (MD) and radial diffusivity (RD) values in the CST. It suggests that applying HF-rTMS over the bilateral leg area of M1 (M1-LEG) is beneficial for SPG11-HSP. In this study, we demonstrate the potential of rTMS to promote neurological recovery from both functional and structural perspectives. It may provide a clinical rationale for using rTMS in the rehabilitation of HSP patients.

## 1. Introduction

Hereditary spastic paraplegia (HSP) comprises a rare group of genetically heterogeneous neurological disorders characterized by lower extremity spasticity and slowly progressive abnormal gait (1). The most common neuroanatomical changes occurring in HSP are the loss of axons in the corticospinal tract (CST) and corpus callosum (CC) (2). Hereditary spastic paraplegia type 11 (SPG11-HSP) is a complex form of HSP mainly due to mutations in the SPG11 gene (3, 4). Although a broader range of neuropathological abnormalities has been reported in complicated HSP, it remains generally accepted that degenerative changes in the long axons of the CST are responsible for motor symptoms of the disease (2, 5).

To date, there is no specific treatment to prevent or reverse the neuron degeneration of HSP, nor is there a specific drug to cure it (3). Conventional therapies such as physical therapy, antispasticity medications and orthopedic braces are still mainstream treatments for motor symptoms in patients with all types of HSP (6). Unfortunately, the results are often less than satisfactory (4). Repetitive transcranial magnetic stimulation (rTMS) is a non-invasive neuromodulation technique that delivers repetitive magnetic pulses at specific cortical sites to modulate motor cortical excitability and improve motor performance through corticospinal projections (7, 8). It is expected to be a promising therapeutic tool for neurodegenerative diseases (9). However, no previous studies have applied rTMS to treat motor symptoms in SPG11-HSP.

Here, we report a case of SPG11-HSP with lower extremity spasticity and gait instability, which were improved by applying high-frequency rTMS (HF-rTMS) at the primary motor cortex (M1).

## 2. Case description

### 2.1. Patient

A 21-year-old Chinese female presented to Shanghai No. 3 Rehabilitation Hospital with a history of progressive lower extremity weakness and gait instability for over 6 years. She showed no abnormalities in motor learning or motor performance in early childhood. Only after age 15 did her motor deficits slowly manifest. The initial manifestation was a slight dragging of the left foot when walking with a tendency to fall, followed by weakness in both lower extremities. The patient was initially seen in pediatrics and underwent traditional physical rehabilitation, but her symptoms slowly worsened. When the patient arrived at our hospital, her balance and walking ability were so poor that she could not take care of herself well. Then, we performed a neurological examination. The mental status was normal (Mini-Mental State Examination score = 28). The examination revealed hyperreflexia of the knee, positive bilateral Babinski's sign. She had a spastic gait with marked lower extremities hypertonia, and her bilateral lower extremity muscle strength was level 4/5. She also had somatosensory deficits, evidenced by impairment of the two-point discrimination threshold and absence of vibration in the distal lower extremity.

**Figure 1:**
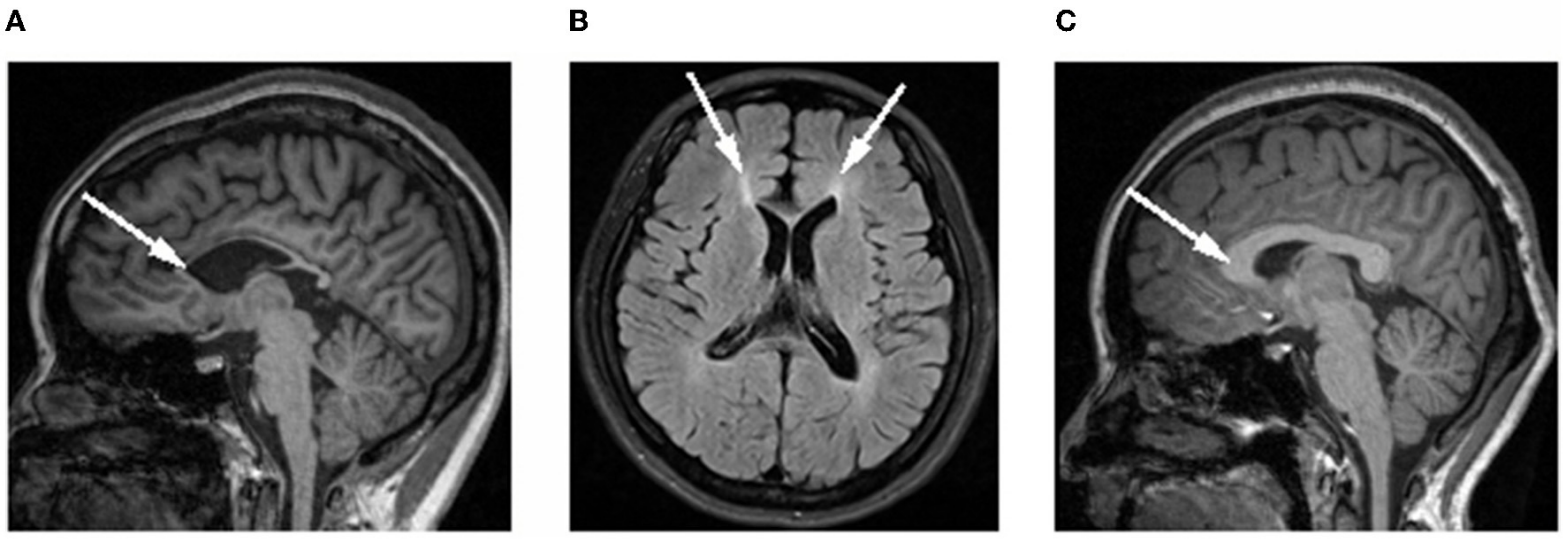
Brain MRI in the patient and a healthy control subject (the same age girl). **(A)** Sagittal T1-weighted image showing thin corpus callosum (white arrow) in the patient, compared to normal corpus callosum morphology in a control subject. **(B)** Axial FLAIR image showing typical “ears of the lynx” sign (white arrow). **(C)** Brain image in the healthy control subject.

Magnetic resonance imaging (MRI) of the brain revealed a markedly thin CC, particularly in the genu and anterior portion of the body, best seen on sagittal T1-weighted image (Figure 1A). Axial FLAIR image (Figure 1B) showed the focal thinning in the genu fibers of the CC, which is referred to as the “ears of the lynx” sign and is a highly correlated imaging presentation with SPG11-HSP (10). The genetic testing identified 2 heterozygous mutations on the SPG11 gene. The heterozygous mutations were located on 2 alleles, one from her mother (chr15:44888407, c.4307_4308del, p.Q1436fs) and the other from her father (chr15:44949428, c.733_734del, p.M245fs), constituting a compound heterozygous mutation. Therefore, the patient was diagnosed with SPG11-HSP.

### 2.2. Treatment approaches

After signing an informed consent form, the patient received HF-rTMS treatment. Every session consisted of 40 trains of stimulation, each lasting 6 s and an intertrain interval of 8 s with a frequency of 5 Hz. There were 1,200 pulses in each session. The stimulation intensity was set at 90% of the resting motor threshold (RMT), and the stimulation sites were the bilateral leg area of M1 (M1-LEG). A total of 2,400 pulses (1,200 on the left side and 1,200 on the right side) were applied daily. We used a figure-8-shaped coil connected to the magnetic stimulator (M-100 Ultimate, Yingchi, Shenzhen, China). The location of the coil was determined by the international 10-20 system, with M1-LEG located ~1–2 cm posterior to the vertex (11). The center of the figure-8-shaped coil was placed 1 cm posterior to the vertex and 1–2 cm laterally on the target hemisphere (11). The coil handle was then pointing medially toward the non-target hemisphere to generate a medial-lateral current in M1-LEG (12). We used the medial-lateral coil orientation because it is proven more effective for targeting lower extremity muscles (12). The patient had undergone the treatment 5 days a week for 5 consecutive weeks without adverse events. During the rTMS treatment period, the existing conventional therapies (physical therapy, ankle-foot orthosis) were continuously used.

### 2.3. Clinical assessment and results

Clinical measures were conducted at baseline (T0) and weekly for 5 weeks during treatment. The Modified Ashworth Scale (MAS) scores were evaluated in 3 joints of the patient's lower extremity with a 6-point (0, 1, 1+, 2, 3, and 4) scale: ankle (dorsiflexion and plantar flexion), knee (extension and flexion), and hip (abduction and adduction). The scores for each joint were recorded, and the scores of the three joints were then summed to calculate a composite score for each leg. The Berg Balance Scale (BBS) consists of 14 tasks, including sitting, standing, and moving around. For the Timed Up and Go (TUG) test, we asked the patient to stand up from an armchair, walk 3 m, turn around, walk backwards, and sit down to the same chair. For performing the 10-meter Walk Test (10 MWT), the patient was instructed to walk in a straight line wearing shoes. The start, 2 m, 8 m and the end of this route were marked on the floor with brightly colored tape. We recorded only the middle 6 m in order to minimize the effect of acceleration and deceleration, disregarding the initial 2 m and the final 2 m of the path (13). Three consecutive measurements were done for the TUG test and 10 MWT, and the average time (in seconds) required to complete the measurements was calculated.

As shown in Table 1, the MAS scores of both legs gradually decreased, and the BBS scores gradually increased with prolonged treatment. Besides, the TUG test and 10 MWT were reduced to varying degrees after treatment.

**Table 1:** Comparison of MAS in the bilateral leg, BBS, 10 MWT and TUG test scores of the patient among 5 weeks.

**Item**		**T0**	**T1**	**T2**	**T3**	**T4**	**T5**
MAS_R	Hip	2.5	2.5	2	1	1	1
	Knee	4	3.5	3.5	3	2.5	2
	Ankle	5	4.5	3.5	3.5	2.5	2.5
	Total (leg)	11.5	11	9	7.5	6	5.5
MAS_L	Hip	2.5	2.5	2	2	1	1
	Knee	4	3.5	3	3	2.5	2.5
	Ankle	5	4.5	3.5	2.5	2.5	2.5
	Total (leg)	11.5	11	8.5	7.5	7	6
BBS		29	33	36	42	45	46
10 MWT (s)		78.63	81.27	85.33	69.14	68.09	70.10
TUG test (s)		139.02	127.05	129.12	105.18	100.48	89.34

MAS, modified Ashworth scale; R, right; L, left; BBS, Berg balance scale; TUG, timed up and go; 10 MWT, Ten-meter walking test; T0, baseline; T1, the first week; T2, the second week; T3, the third week; T4, the fourth week; T5, the fifth week.

### 2.4. TMS assessment and results

Neurophysiological changes were assessed using single-pulse TMS (11). The patient was comfortably seated in a chair for the assessment. The assessor placed the coil tangentially on the M1 based on the international 10–20 system. Indicators related to the hand area of M1 (M1-HAND) and the M1-LEG were assessed separately. Electromyogram signals were acquired using Ag/AgCl surface electrodes (Kendall Medi-Trace) on the bilateral first dorsal interosseous (FDI) and tibialis anterior (TA) muscles, respectively. RMT was defined as the minimum TMS intensity evoking motor evoked potential (MEP) with amplitudes at least 50 μV in 5 out of 10 consecutive single-pulse stimuli while the target muscle was relaxed (14). RMT values were expressed as a percentage of the maximum stimulator output (%MSO). We consecutively evoked MEPs with 120% RMT and calculated the average latency values and peak-to-peak values of 10 MEPs. Thereafter, the peripheral motor latency from the 7th cervical magnetic stimulation was subtracted from the MEP cortical latency to obtain the central motor conduction time (CMCT) (15). Separately, we instructed the patient to maintain a tonic contraction of 20% maximal voluntary contraction of the FDI and measured cortical silent period (CSP) by stimulating the contralateral M1-HAND at 120% RMT intensity.

We evaluated the patient twice to compare the outcomes before and after HF-rTMS treatment. Table 2 shows an increase in MEP amplitude and a decrease in RMT in the upper and lower extremities compared to pre-treatment, a slight reduction in CMCT in the upper extremities, and a significant decline in the difference in CSP between the left and right hemispheres.

**Table 2:** The changes in cortical excitability of the patient before and after treatment.

**Cortical excitability**		**Pre-treatment**	**Post-treatment**	**Difference**
M1-HAND	Right RMT (%MSO)	70	64	6 (↓)
	Right MEP amplitude (μV)	50	307	257 (↑)
	Right CMCT (ms)	9.7	8.6	1.1 (↓)
	Right CSP (ms)	36.1	94	57.9 (↑)
	Left RMT (%MSO)	66	64	2 (↓)
	Left MEP amplitude (μV)	150	512	362 (↑)
	Left CMCT (ms)	9.3	8.8	0.5 (↓)
	Left CSP (ms)	132.7	114	18.7 (↓)
M1-LEG	Right RMT (%MSO)	85	80	5 (↓)
	Right MEP amplitude (μV)	75	117	42 (↑)
	Left RMT (%MSO)	77	68	9 (↓)
	Left MEP amplitude (μV)	124	233	109 (↑)

M1-HAND, hand area of the primary motor cortex; M1-LEG, leg area of the primary motor cortex; RMT, resting motor threshold (in % of the maximal stimulator output); MEP amplitude, motor-evoked potential (in microvolts); CMCT, central motor conduction time (in milliseconds); CSP, cortical silent period (in milliseconds).

### 2.5. DTI assessment and results

DTI data were collected using a 3-Tesla MRI scanner (SIEMENS VERIO, Erlangen, Germany) with an 8-channel head coil. To better understand the imaging changes, a healthy female volunteer of the same age was recruited as a control. The patient was scanned twice (pre-treatment and post-treatment) and the healthy control subject was only scanned once. The same DTI acquisition parameters were used for the patient and the healthy control subject as follows: acquisition matrix size = 128 × 128, field of view (FOV) = 240 mm × 240 mm, repetition time (TR) = 10,000 ms, echo time (TE) = 89 ms, flip angle = 90°, direction = 62, b = 0, 1,000 s/mm^2^, slice thickness = 2 mm and slice gap = 0.

All DTI data were preprocessed using FSL software (http://www.fmrib.ox.ac.uk/fsl). Then, whole-brain deterministic fiber tracking was processed based on fiber assignment by continuous tracking (FACT) algorithm and performed using Diffusion Toolkit and TrackVis (http://www.trackvis.org). The white matter fiber tracking was terminated when the maximum turning angle > 45° or fractional anisotropy (FA) < 0.2. After the whole-brain fiber tracts were obtained, the CST were traced with the fiber tracts passing through cerebral peduncles, the posterior extremity of the internal capsule and M1 as the regions of interest (ROIs) were obtained by manual segmentation. Finally, the mean value of FA, mean diffusivity (MD) and radial diffusivity (RD) were extracted from each CST. Besides, region-based analysis of the CC was applied using atlas-based predefined brain regions on the basis of the John Hopkins University (JHU) white matter tractography atlas (16).

**Figure 2:**
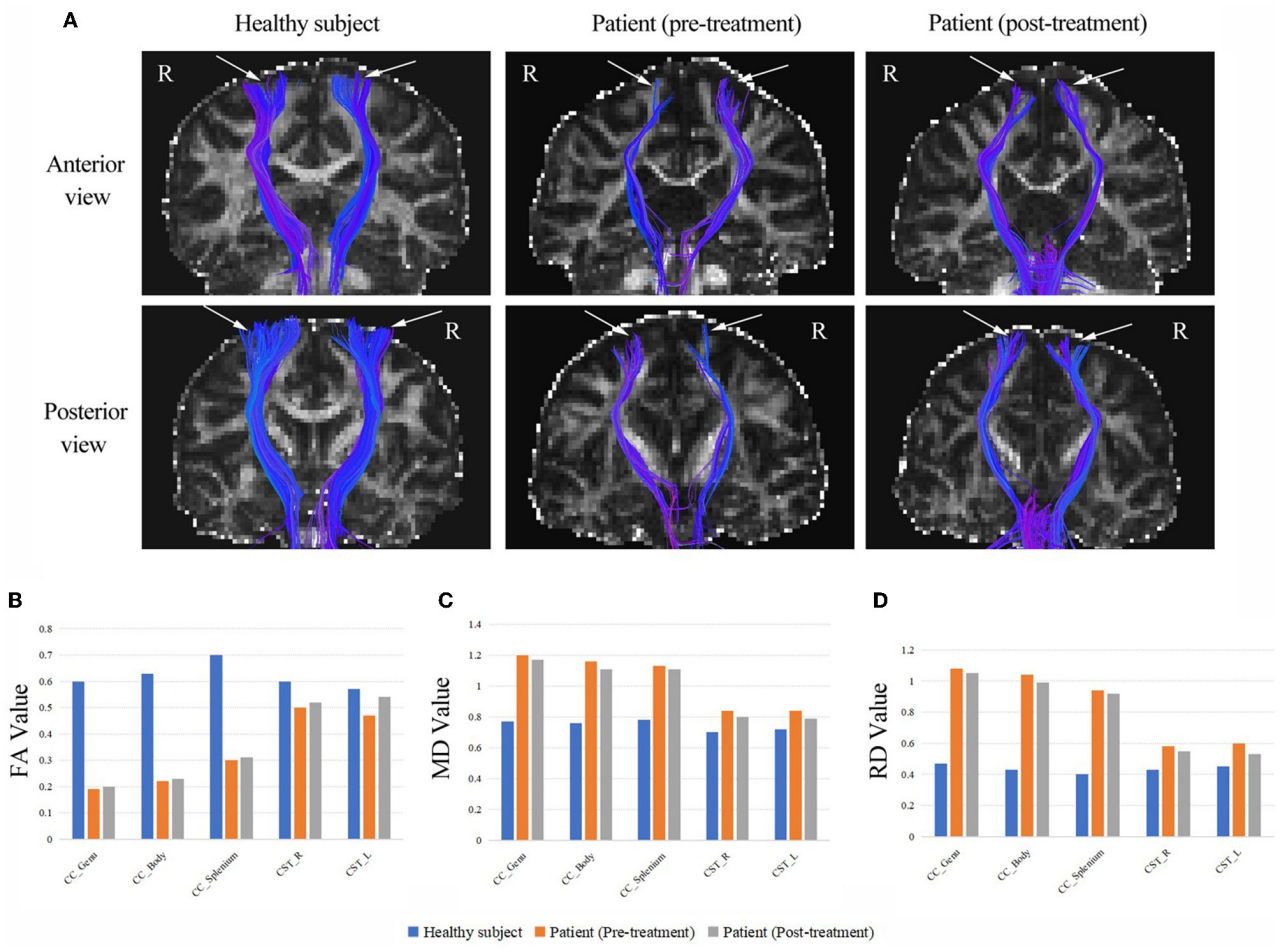
**(A)** The CSTs of the patient (pre-treatment and post-treatment) and a healthy subject (the same age girl). White arrows are used to highlight the differences. The DTI revealed thinning of the CSTs in bilateral hemispheres compared to those of a healthy control subject. **(B)** The FA values from CC and CST of the patient (pre-treatment and post-treatment) and the healthy subject. **(C)** The MD values from CC and CST of the patient (pre-treatment and post-treatment) and the healthy subject. **(D)** The RD values from CC and CST of the patient (pre-treatment and post-treatment) and the healthy subject. CC, corpus callosum; CST, corticospinal tract; FA, fractional anisotropy (10^−3^ mm^2^/s); MD, mean diffusivity (10^−3^ mm^2^/s); RD, radial diffusivity (10^−3^ mm^2^/s); R, right; L, left.

As shown in Figure 2, DTI revealed thinning of the CSTs in bilateral hemispheres compared to those of a healthy control subject. The results showed a marked reduction of FA values and increases in MD and RD values in the CSTs, which indicated a loss of integrity and density of the CSTs. We also found a drastically reduced FA values and increased MD and RD values throughout the CC of the patient, following the observed extreme thinning of the CC areas in the brain MRI. Fortunately, the patient had increased FA values and decreased MD and RD values in the CST and CC after treatment. A detailed description of the FA, MD and RD values from CC and CST of the patient (pre-treatment and post-treatment) and the healthy subject, and the difference between pre-treatment vs. post-treatment and the difference between patient vs. healthy control as a percentage change in FA, MD and RD are provided in Supplementary material.

## 3. Discussion

To our knowledge, this is the first report of HF-rTMS for SPG11-HSP and the first report of a Chinese patient. Before rTMS treatment, the patient received conventional therapies including physical therapy to stretch the spastic muscles and an ankle-foot orthosis to prevent foot drop. However, the patient's walking ability did not improve. In addition, the patient refused to take oral antispasticity medication due to the inability to tolerate the side effects. Therefore, rTMS, an approach targeting central nervous system modulation, played a crucial role in improving the symptoms. In this study, we revealed the recovery of CST and improvement of motor function after HF-rTMS treatment using TMS and DTI assessment. This disease is rare and incurable, and our findings have important implications for rehabilitation programs to treat SPG11-HSP.

The patient showed significant improvement in spasticity, according to MAS. In the lower extremity, the pathophysiology of spasticity is the reduction of reciprocal inhibition that plays a major role (17, 18). Previous studies demonstrated that the reduction of this spinal inhibitory mechanism is involved in the occurrence of spasticity in HSP patients (19, 20). Spinal cord stimulation can improve the spasticity of the lower extremity in patients with HSP (21). Another study found that rTMS enhances descending projections between the motor cortex and spinal inhibitory circuits, which contributes to the reduction of lower extremity spasticity (22). Perez et al. also demonstrated that rTMS at 5 Hz over M1-LEG could modulate transmission in specific spinal cord circuits through changes in the corticospinal drive (23). Therefore, we theorize that cortical and spinal cord neural remodeling associated with relief of spasticity has occurred in our patient, facilitated by rTMS.

In addition, our patient showed promising improvements in balance and walking ability according to BBS, TUG test and 10 MWT. It is not only due to reduced lower extremity spasticity but also associated with improved postural control. Behaviorally, the TUG test improves more significantly than 10 MWT. As we know, 10 MWT reflects walking speed, while the TUG test emphasizes postural control. Postural control and walking are generally considered voluntary motor functions mainly controlled by subcortical and spinal cord regions (24). However, it has been found that the motor cortex also participates in some aspects of postural control (25). The M1 plays a key role in motor control, including controlling body posture and planning and execution of movements (26). Previous study suggested that delayed postural responses and reduced MEP amplitude of the legs are responsible for balance impairments in HSP patients, and the mechanism may be diminished corticospinal drive onto spinal interneurons (27). Therefore, we believe that HF-rTMS may provide good modulation of postural control by enhancing corticospinal drive.

Other studies revealed that the ongoing modulation of corticospinal excitability and intracortical inhibition within M1 is critical for successful restraint and cancellation of actions (28, 29). In addition to activating corticospinal neurons, rTMS activates intracortical inhibitory and excitatory neural circuits in the M1 (30). GABAergic inhibitory brain circuits are important to motor control (31). TMS-invoked CSP is an index of GABAB-mediated intracortical inhibition within M1 (32), and the duration of CSP reflects individual inhibitory control capacities (33). Because of substantial interindividual variability, the relative right-to-left difference in CSP is generally considered clinically significant (11). Our patient had a significantly lower duration of CSP in the right hemisphere than in the left before treatment. It suggested that the inhibitory interneurons in the right hemisphere are more damaged, which is consistent with a high RMT in the right hemisphere. Therefore, the patient also presented clinically with weaker control in the left leg than in the right leg. After treatment, the improvement in postural control was accompanied by a significant reduction in CSP differences between the left and right hemispheres. It showed that the corticospinal excitatory and inhibitory regulation tends to be balanced, which may also be one of the favorable factors for the recovery of her balance and walking ability. However, the details of the corticospinal inhibition mechanisms affecting motor inhibition function cannot be determined by examining the silent period alone. Movement coordination relies on both the activation of each hemisphere and the communication between hemispheres mediated via the CC. Furthermore, the mechanism of walking is complex. In addition to central control, factors such as sensory and spatial cognitive function can also impact walking. The underlying mechanisms require further exploration.

The coordinated control of the upper and lower extremity is an important factor in adjusting posture and maintaining human balance during walking (34, 35). We considered that the function of the upper extremity also affects the recovery of walking ability. Thus, in addition to the neurophysiological assessment of the lower extremity, we also assessed the upper extremity. After treatment, the patient had an increased MEP amplitude and a decreased RMT for both upper and lower extremities. The decreased RMT reflects increased excitability of the most sensitive group of neurons in the stimulated area in M1 (36). The amplitude of MEP represents the global excitability of cortical interneurons, corticospinal neurons and spinal motoneurons (37). After HF-rTMS treatment, the neuronal excitability of M1-LEG increased, reaching the target muscle via the conduction pathway and facilitating the recovery of function in the patient's lower extremities. Meanwhile, the MEP amplitude measured through the FDI muscles of the upper extremity also increased, even though the patient did not exhibit signs of upper extremity dysfunction. It indicates that the motor neurons and motor pathways associated with the upper extremity muscles were activated, which may make the upper extremity motions more flexible and indirectly create favorable conditions for improving the patient's walking stability. Previous studies suggested that the activity motor cortical areas might improve gait by inducing activity modulation of spared descending motor pathways (38, 39). Furthermore, a slight decrease in CMCT in the upper extremities compared to the pre-treatment indicates an increase in the conduction velocity of upper motor neurons, which may reflect improved axonal performance in the fast-conducting fibers of corticospinal neurons (40). The above results indicate that the patient's motor cortex and the motor conduction pathways were activated bilaterally after a series of treatments, providing a basis for functional remodeling of the nervous system.

Moreover, we used the DTI metrics to analyze the microstructural alterations in the CSTs before and after treatment, including FA, RD and MD, which can characterize axonal injury and neuronal fiber myelination by quantifying white matter damage (41). A previous study has found that patients with HSP have lower FA values and higher RD values in the CST compared to healthy subjects (42), which is consistent with our results. It indicated the impaired structural integrity of the CST in the patient with axonal damage and neuronal fibers demyelination. However, we are pleased to observe an increase in FA values and a decrease in MD and RD values in the CST at the patient's second DTI scan (i.e., after treatment). Additionally, these indicators were similarly altered in the genu, body and splenium parts of the CC. A study confirmed that HF-rTMS promotes motor recovery while improving diffusion microstructures in motor-related white matter and gray matter brain regions, as evidenced by a significant increase in FA values of the CST (43). So, the changes in DTI metrics demonstrated that the restoration of white matter fiber connections occurred in the motor conduction pathways, providing a basis for the patient's functional recovery.

There are some limitations in generalizing the effects of rTMS based on this study. Since it is a case report, we can't completely rule out that other factors affected the results. Additionally, it is a qualitative report with no statistical analysis. In addition, the whole-brain effects induced by HF-rTMS should be considered in the future. Besides, there was a lack of follow-up in this case. Due to the health crisis of COVID-19, the patient did not return for a follow-up evaluation. We conducted a telephone interview after 4 months of treatment and learned that the patient's ambulation was maintained for 1–2 months after discharge and then tended to decline. Therefore, the long-term beneficial effects of HF-rTMS treatment could not be determined, and this should be investigated in depth in future studies.

## 4. Conclusions

In summary, this case report offers a new rehabilitation prescription. We demonstrate the potential of this strategy to promote motor nervous system recovery in SPG11-HSP patients from a functional and structural perspective. We expect researchers can provide additional clinical evidence of the benefits of HF-rTMS applied to the M1-LEG to improve walking ability and reduce spasticity in patients with HSP.

## Data availability statement

The original contributions presented in the study are included in the article/Supplementary material, further inquiries can be directed to the corresponding authors.

## Ethics statement

Written informed consent was obtained from the individual(s) for the publication of any potentially identifiable images or data included in this article.

## Author contributions

SC and ZZ designed and conceptualized the study, treated the patient drafted, and finalized the manuscript. CS and WL supervised the progression and revised the manuscript. XC and SZ assisted in obtaining the TMS assessment. MR collected and sorted out the related materials. SX helped with DTI data processing. XS and XiaZ analyzed and interpreted the data. XinZ was responsible for the physical therapy. All authors read and approved the submitted version.
