# Supplementary material for: Case report: High-frequency repetitive transcranial magnetic stimulation for treatment of hereditary spastic paraplegia type 11
Source: Front Neurol. 2023 May 18;14:1162149. doi: 10.3389/fneur.2023.1162149 (PMC10232891; doi:10.3389/fneur.2023.1162149)
Supplement: Supplementary file 1 [file Data_Sheet_1.pdf]

Supplemental materials

Table S1. FA, MD and RD values from CC and CST of the patient (pre-treatment and post-treatment) and the healthy subject. The difference between pre-treatment vs. post-treatment and difference between patient vs. healthy control as a percentage change in FA, MD and RD. The DTI results with marked reductions of FA and increases in MD and RD values in the patient in the CSTs. The indications of loss of the CSTs integrity and density by DTI are consistent with the patient's presentation of lower extremity motor deficits. We also found a drastically reduced FA and marked increase in MD and RD values throughout the CC, following the observed extreme thinning of the CC areas in brain MRI. Fortunately, the patient had increased FA values and decreased MD and RD values in the CST and CC after treatment.

	The patient						The healthy subject			The percentage change					
	Pre-treatment			Post-treatment						pre-treatment vs. post-treatment [(post-pre)/pre,%]			patient vs. healthy control [(pre-healthy)/healthy,%]		
Fiber bundles	FA	MD	RD	FA	MD	RD	FA	MD	RD	FA	MD	RD	FA	MD	RD
CC_Genu	0.19	1.20	1.08	0.20	1.17	1.05	0.60	0.77	0.47	5.26	-2.50	-2.78	-68.33	55.84	129.79
CC_Body	0.22	1.16	1.04	0.23	1.11	0.99	0.63	0.76	0.43	4.55	-4.31	-4.81	-65.08	52.63	141.86
CC_Splenium	0.30	1.13	0.94	0.31	1.11	0.92	0.70	0.78	0.40	3.33	-1.77	-2.13	-57.14	44.87	135.00
CST_R	0.50	0.84	0.58	0.52	0.80	0.55	0.60	0.70	0.43	4.00	-4.76	-5.17	-16.67	20.00	34.88
CST_L	0.47	0.84	0.60	0.54	0.79	0.53	0.57	0.72	0.45	14.89	-5.95	-11.67	-17.54	16.67	33.33

CC, corpus callosum; CST, corticospinal tract; FA, fractional anisotropy; MD, mean diffusivity [10-3 mm2/s]; RD, radial diffusivity [10-3 mm2/s]; R, right; L, left.
